# Systematic review of the efficacy and safety of antiretroviral drugs against SARS, MERS or COVID‐19: initial assessment

**DOI:** 10.1002/jia2.25489

**Published:** 2020-04-01

**Authors:** Nathan Ford, Marco Vitoria, Ajay Rangaraj, Susan L Norris, Alexandra Calmy, Meg Doherty

**Affiliations:** ^1^ Department of HIV, Hepatitis and Sexually Transmitted Infections World Health Organization Geneva Switzerland; ^2^ Science Division Quality of Norms and Standards Department World Health Organization Geneva Switzerland; ^3^ HIV/AIDS Unit Division of Infectious Diseases Geneva University Hospitals Geneva Switzerland

**Keywords:** antiretroviral therapy, HIV, MERS, SARS, coronavirus, COVID‐19

## Abstract

**Introduction:**

Several antiretroviral drugs are being considered for the treatment of COVID‐19, the disease caused by a newly identified coronavirus, (SARS‐CoV‐2). We systematically reviewed the clinical outcomes of using antiretroviral drugs for the prevention and treatment of coronaviruses and planned clinical trials.

**Methods:**

Three databases were screened from inception to 30 March 2020 for studies reporting clinical outcomes of patients with SARS, MERS or COVID‐19 treated with antiretrovirals.

**Results:**

From an initial screen of 433 titles, two randomized trials and 24 observational studies provided clinical outcome data on the use of antiretroviral drugs; most studies reported outcomes using LPV/r as treatment. Of the 21 observational studies reporting treatment outcomes, there were three studies among patients with SARS, six studies among patients with MERS and 12 studies among patients with COVID‐19. In one randomized trial 99 patients with severe COVID‐19 illness were randomized to receive LPV/r (400/100 mg twice a day) and 100 patients to standard of care for 14 days: LPV/r was not associated with a statistically significant difference in time to clinical improvement, although LPV/r given within 12 days of symptoms was associated with shorter time to clinical improvement; 28 day mortality was numerically lower in the LPV/r group (14/99) compared to the control group (25/100), but this difference was not statistically significant. The second trial found no benefit. The certainty of the evidence for the randomized trials was low. In the observational studies 3 out of 361 patients who received LPV/r died; the certainty of evidence was very low. Three studies reported a possible protective effect of LPV/r as post‐exposure prophylaxis. Again, the certainty of the evidence was very low due to uncertainty due to limited sample size.

**Conclusions:**

On the basis of the available evidence it is uncertain whether LPV/r and other antiretrovirals improve clinical outcomes or prevent infection among patients at high risk of acquiring COVID‐19.

## 1 INTRODUCTION

Several antiretroviral drugs are being considered for use in the treatment of COVID‐19, the disease caused by a newly identified coronavirus, (SARS‐CoV‐2). Protease inhibitors have been considered as candidate therapy because they inhibit enzymes that activate envelope glycoproteins as part of the process of viral entry into cells [1]. The use of lopinavir/ritonavir (LPV/r) has been supported by data from in vitro studies, animal models and positive clinical outcomes when LPV/r was given to patients infected with severe acute respiratory syndrome (SARS) and Middle East Respiratory Syndrome (MERS) diseases also caused by coronaviruses [2, 3, 4, 5]. Other antiretrovirals have been proposed based on virtual screening and in vitro studies, and several clinical trials are planned. Lopinavir/ritonavir (LPV/r) is included in rapid guidance issued by researchers from Wuhan University based on clinical use during prior epidemics of severe acute respiratory syndrome (SARS) and MERS coronavirus (CoV) infections [6].

This systematic review summarizes the clinical outcomes of using antiretroviral drugs for the prevention and treatment of coronaviruses and planned clinical trials.

## 2 METHODS

Based on in vitro activity, molecular docking studies, or reported use in prior reviews the following drugs were screened [7, 8, 9, 10, 11]: lopinavir/ritonavir, emtricitabine, tenofovir, atazanavir, ritonavir, darunavir, nelfinavir, indinavir, saquinavir, lamivudine and zidovudine (Search strategy provided in Appendix S1).

Three databases – Medline via PubMed, EMBASE and the Cochrane Library – were screened from inception to 30 March 2020 for studies reporting clinical outcomes of patients with SARS, MERS or COVID‐19 treated with antiretrovirals; studies using antiretrovirals for the prevention of these infections were also sought. The WHO database of publications on COVID‐19 was also searched https://www.who.int/emergencies/diseases/novel-coronavirus-2019/global-research-on-novel-coronavirus-2019-ncov.

Any study design that reported clinical outcome data was included, and there were no language restrictions. Clinicaitrials.gov and Chictr.org.cn were searched for ongoing and completed trials. Data are summarized per study, but not pooled in meta‐analysis due to the limited number of studies reporting outcomes for each disease. The review was conducted by a single reviewer (NF), with data extraction validated by a second reviewer (AR). The quality (or certainty) of the evidence was assessed using the Grading of Recommendations, Assessment, Development and Evaluation (GRADE) approach [12].

## 3 RESULTS AND DISCUSSION

### 3.1 Antiretroviral drugs for treatment

From an initial screen of 433 titles, two randomized controlled trials and 21 observational studies provided clinical outcome data on the use of antiretroviral drugs for treatment, and 3 studies reported outcomes for prevention. Three studies were excluded: one because cause of infection was unclear [13], one because the original study was retracted during the conduct of this systematic review [14] and one because lamivudine was given to control chronic hepatitis B infection and its use could not be linked to SARS outcomes [15]. Among the included studies, the majority reported outcomes using LPV/r as treatment; two two studies reported outcomes among HIV‐positive individuals who were on a combination antiretroviral drugs for management of HIV [16, 17].

Characteristics of included studies and patient outcomes are summarized in Table 1.

**Table 1:** Clinical studies evaluating LPV/r for MERS, SARS and Covid‐19

Author Country	Population Study design	Intervention	Co‐interventions	Timing/duration of therapy	Comparitor	Mortality	Details
Treatment							
SARS							
Chan 2003 [2] China	75 adults Matched cohort study	LPV/r 400/100 Q12H + standard treatment protocol	Ribavarin either as cotreatment with LPV/r or as rescue therapy, pulse Methylprednisone 3 mg/kg/day or tailing hydrocortisone therapy 21 days 100 to 200 mg/day + mechanical ventilation if required	10 to 14 days depending on severity	977 matched controls from hospital data	LPV/r: 5/75 died Control: 147/977 died	Reduction in mortality: 2.3% (0% to 6.8%) vs. 15.6% (9.8% to 22.8%) Reduction in intubation rate: 0% vs 11% (7.7% to 15.3%)
Chu 2004 [18] China	41 adults Case‐control study with historical controls	LPV/r 400/100 Q12H as initial therapy (n = 12), time of onset of symptoms 3.5 days. For rescue treatment (n = 29) time of onset of symptoms 14 days	Ribavarin and IV steroids	14 days	111 historical controls	LPV/r: 0/41 died Control: 7/111 died	Treatment group: 21‐day mortality/ARDS: 0/41, ARDS/death before 21 days: 1/44; Historical controls: 21 day mortality/ARDS: 7/111, ARDS/death before 21 days: 32/111
Wong 2004 [17] China	30‐year‐old man Case report	Abacavir 300 mg Q12H, efavirenz 600 mg once daily, TDF 300 mg Q12H, LPV/r 4 x 133.3 mg/33.3 mg	Ribavirin 1200 mg three times a day and prednisolone 25 mg three times a day 3TC (for hepatitis flare)	ARVs provided for HIV treatment	n/a	0/1 died	Recovered
MERS							
Spanakis 2014 [19] Greece	69‐year‐old man Case report	LPV/r 400/100 Q12H	peg‐interferon 180 mcg 1/wk for 12 days, RBV, empirical antibiotics	2 months and 6 days; RBV d/c on day 20	n/a	LPV/r: 1/1 died	Died due to Septic Shock + MODS; incidental diagnosis of adenocarcinoma colon
Meyer 2015 [20] Austria	29‐year‐old woman Case report	LPV/r	Supportive intensive care therapy	nr	n/a	LPV/r 0/1 died	Complete clinical recovery
Shalhoub 2015 [16] Saudi Arabia	51‐year‐old man Case report	TDF/FTC 300/200 mg once daily + ATV/r 300 mg/100 mg) once daily	Supportive intensive care therapy IFN 2a 180 mcg 1/wk, RBV (loading dose of 2 gm, followed by 600 mg orally every 12 hours) Treatment for CMV prophylactic trimethoprim/sulfamethoxazole 960 mg daily	ARVs initiated for HIV treatment	n/a	0/1 died	Recovered
Kim 2016 [22] Rep Korea	64‐year‐old man Case Report	LPV/r 400/100 Q12H	Ribavarin 2 g LD, 1.2 g TID, IFN 2alpha 180 mcg/0.5 mL from day 4 of admission, Empirical therapy with piperacillin/tazobactam and azithromycin from Day 1 of admission	7 days	n/a	LPV/r: 0/1 died	Discharged on day 13 due to clinical improvement
Choi 2016 [3] Rep Korea	120 adults Retrospective observational study	138 patients received antivirals among whom 120 received LPV/r‐containing regimens	Antibiotics, haemodialysis, ECMO and convalescent sera. >80% of patients given LPV/r also received IFN	Median time from onset of illness to treatment was 6 days	n/a	LPV/r: 24/120 died	Median interval from symptom onset to death was 14 days
Alhumaid 2018 [21]^a^ Saudi Arabia	41 patients Retrospective observational study	41 patients received LPV/r	IFN, RBV and antibiotics	nr	n/a	LPV/r 17/41 died	
COVID‐19							
Cao 2020 [23] China	199 patients Randomized trial	100 adult patients received LPV/r 400/100 Q12H	Supportive care	14 days	Supportive care alone	LPV/r 14/99 died Control 25/100	LPV/r not associated with a statistically significant difference in time to clinical improvement
Li 2020 [24] China	21 adult patients received LPV/r Randomized trial	LPV/r 200/500 Q12H	Some patients received gama globulin. All patients received supportive care and oxygen therapy if needed	7 to 14 days	16 received arbidol 7 received no antivirals	LPV/r 0/21 died	Mild/moderate cases enrolloed. More patients treated with LPV/r progressed to severe/critical status
Wang 2020 [28] China	4 adult patients Case series	LPV/r 400/100 Q12H	Umifenovir (Arbidol), SFJDC	6 to 15 days	n/a	LPV/r: 0/3 died	Outcome of 1 patient unknown
Lim 2020 [25] Rep Korea	54‐year‐old man Case report	LPV/r 400/100 Q12H from day 8 of admission, day 10 from onset of symptoms	Other treatments included: Azithromycin, ceftriaxone, levofloxacin/ Tazobactam and 1 dose of Peramivir	10 days	n/a	LPV/r: 0/1 died	Patients showed clinical improvement following initiation with LPV/r
Han 2020 [26] China	47‐year‐old man Case report	LPV/r 400/100 daily on day 4 of illness	Methylprednisolone (40 mg daily), IFN alfa‐2b (10 million IU daily), ambroxol hydrochloride (60 mg daily) and moxifloxacin hydrochloride (0.4 g daily	Unclear, but discharged after 10 days	n/a	LPV/r: 0/1 died	Patient received LPV/r and was discharged on day 10.
Kim 2020 [27] Rep Korea	35‐year‐old woman Case report	LPV/r 800/200 daily	Oxygen supplementation	Unclear but fever persisted for 10 days	n/a	LPV/r: 0/1 died	
Young 2020 [29] Singapore	5 adults Retrospective cohort	5 patients treated with LPV/r (200 mg/100 mg Q12H for up to 14 days)	Oxygen supplementation	within 1 to 3 days of desaturation	n/a	LPV/r: 0/5 died 3/5 improved 2/5 developed progressive respiratory failure	4/5 patients developed nausea, vomiting, and/or diarrhoea, and 3 developed Abnormal liver function test results. Only 1 completed the full 14‐day treatment course
Chen 2020 [31] China	99 patients, of which 75 received LPV/r Retrospective cohort	LPV/r 500 mg Q12H	oseltamivir (75 mg every 12 hours, orally), ganciclovir (0·25 g every 12 hpurs, intravenously). Antibiotics	3 to 14 days	n/a	2/75 died	57 remained in hospital 31 discharged 11 died
Jun 2020 [32] China	52 patients received LPV/r Retrospective cohort	LPV/r Q12H for 5 days	IFN alpha‐2b and supportive care		ArdiboL: 34 patients No antivirals: 48 patients	LPV/r: 0/52	No reported deaths LPV/r: 2/52 severe Abidol: 1/33 Control: 2/48
Liu 2020 [30] China	10 patients received LPV/r Retrospective cohort	LPV/r 400/100 Q12H	Oxygen supplementation. I patient also received TDF for underlying liver disease. 9/10 also received IFN alpha‐2b	5 days from onset of symptoms	n/a	LPV/r: 0/10	
Deng 2020 [33] China	33 patients received LPV/r Retrospective cohort	LPV/r 400/100 Q12H	Some patients received corticosteroids Supportive care	5 to 21 days	16/33 patients also received arbidol	LPV/r: 0/17 LPV/r/arbidol: 0/16	After 14 days, coronavirus no longer detected by PCR
Liu 2020Liu 2020 [34] China	56 patients, of which 53 patients received LPV/r Retrospective cohort	LPV/r 400/100 Q12H	Some patients received IFH & traditional Chinese medicines		n/a	3/56 Unclear Who received LPV/r	Outcomes not linked to receipt of LPV/r
Wan [35] China	135 adult patients Retrospective cohort	LPV/r (dose not reported)	All received interferon Some received corticosteroids and traditional Chinese medicine	nr	n/a	LPV/r 1/135	Patient who died considered severe case
Cai [36] China	45 patients received LPV/r Comparative cohort study	LPV/r 400/100 Q12H	IFN‐α1b 60 μg twice daily	14 days	Favipiravir	0/45 died	
Prevention							
Chen 2003 [38] China	19 patients Individuals with HIV (AIDS) infected with SARS Retrospective cohort	11/19 patients received ARVs: D4T/3TC/EFV = 3, d4T/3TC/NVP = 2, d4T/ddI/NVP = 3, Combivir/EFV = 1, Indinavir/EFV = 2	Remaining 8 patients received treatment for opportunistic infections	15 patients stayed for >1 month with SARS patients on the same floor.	n/a	LPV/r: 0/1 infected	All 19 HIV patients (with AIDS) on the floor tested negative for SARS
Park 2019 [39] Rep Korea	123 HCWs with unprotected exposure to a MERS‐CoV case of which 43 had a high‐risk exposure Retrospective case control study	22 received PEP and 21 were not given PEP; PEP protocol was RBV + LPV/r initiated between day 1 and day 3 after last unprotected exposure to the patient	2 HCWs in the non‐PEP group wore masks, 3 HCWs wore gloves as personal protective equipment	PEP given until day 14, initiated within 36 post exposure, median duration of PEP 12 days	Historical controls from 4 hospitals located far apart	LPV/r: 0/22 infected Control: 6/21 infected	6/43 had MERS‐CoV infection; Attack rate in PEP Vs non‐PEP groups: 0% Vs 28.6%, OR: 0.405 (0.274 to 0.599)
Guo 2020 [40] China	8 HIV positive individuals with COVID‐19 disease compared with 1166 without COVID‐19 disease	947 patients received NNRTI‐ regimen 119 received LPV/r‐based regimen	Use of protection measures unknown	All antiretrovirals taken as HIV treatment	HIV/AIDS patients in Wuchang and Qingshan district	LPV/r: 0/8 infected	Results not statistically significant

3TC, lamivudine; ARDS, acute respiratory distress syndrome; ATV/r, ritonavir‐boosted atazanavir; D4t, stavudine; ECMO, extracorporeal membrane oxygenation; HCWs, Healthcare workers; IFN, Interferon alpha; IU, international units; IV, intravenous; LPV/r, boosted lopinavir/ritonavir; MERS, middle‐east respiratory syndrome; MODS, multiple organ dysfunction syndrome; n/a, not applicable; nCoV, novel coronavirus; nr, not reported; NVP, nevirapine; peg‐IFN, pegylated interferon; PEP, post‐exposure prophylaxis; Q12H, twice daily; RBV, Ribavarin; SARS, Severe acute respiratory syndrome; SFJDC, ShuFengJieDu capsule; TDF, tenofovir.

^a^ Additional information provided by the authors.

### 3.2 SARS

Two observational studies and one case report among patients with SARS [2, 17, 18] reported outcomes of patients who were given antiretrovirals. A study from China reported a reduction in mortality in patients receiving LPV/r of 2.3% (95% CI 0% to 6.8%) compared to matched controls (15.6%, 9.8% to 22.8%) [2]. A second study from China reported that none of the 41 patients given LPV/r died compared with seven of 111 patients in the control group [18]. The third study, also from China, was a case report of a 30‐year‐old HIV‐positive man who recovered; he was receiving abacavir, efavirenz, tenofovir and LPV/r as antiretroviral therapy [17]. All patients also received ribavirin and steroids of varying dose and duration.

### 3.3 MERS

Six observational studies, including two retrospective observational studies [3, 21] and four case reports [16, 19, 20, 22] – one was from Greece, one from Austria, two from Saudi Arabia and two from the Republic of Korea – provided data on patients diagnosed with MERS. There were 42 deaths among 165 patients who were given LPV/r together with other interventions including ribavirin and pegylated interferon.

### 3.4 COVID‐19

One randomized, controlled open‐label study reported on the efficacy and safety of LPV/r for treating hospitalized adults with severe COVID‐19 [23]. In this trial 99 patients received LPV/r (400/100 mg twice a day; median time between symptom onset and randomization 13 days) and 100 patients received standard care for 14 days. LPV/r was not associated with a statistically significant difference in time to clinical improvement; 28 day mortality was numerically lower in the LPV/r group (14/99) compared to the control group (25/100), but this difference was not statistically significant in the intention‐to‐treat analysis. Accelerated clinical recovery and reduced mortality were observed in those treated within 12 days of symptom onset, but not in those treated later. Almost half of patients in the LPV/r group (46 patients, 48.4%) and control group (49 patients, 46.7%) reported one or more adverse events: gastrointestinal‐related complaints including nausea, vomiting and diarrhoea were more common in the lopinavir/ritonavir group. A second randomized trial assessed patients admitted to hospital with mild/moderate COVID‐19, and compared outcomes of 21 patients given LPV/r (200mg/50mg twice a day) with 16 patients given ardibol and 7 patients who were not given any antiviral therapy [24]. In this trial, LPV/r did not show any benefit in terms of time to viral clearance (PCR negativity) or progression to severe disease. For both trials, certainty of the evidence was low due to risk of bias (investigators not blinded to the intervention, and imprecision.

In the observational studies, three case reports [25, 26, 27]. three case series [28, 29, 30], and six observational studies [31, 32, 33, 34, 35, 36] reported outcomes of patients with COVID‐19 who received LPV/r; nine studies were from China, one was from Singapore and two from the Republic of Korea. Among the 361 patients in the nine studies where outcomes could be associated with receipt of LPV/r, three patients died. One study reported that 53 of 56 patients received LPV/r and three patients died; however, it was unclear how many of the patients who died had received LPV/r [31].

LPV/r is recommended by WHO as part of second‐line antiretroviral therapy [37]. Among people living with HIV receiving LPV/r diarrhoea, nausea and vomiting are commonly reported side effects at start of treatment [22]. These side effects were reported by four out of five individuals who received LPV/r for the treatment of COVID‐19 in Singapore, and only one individual completed the 14‐day treatment course as a result of adverse events [29].

The certainty of the evidence for outcomes across these three diseases is very low. The sample size was small and only two studies provided comparative outcomes (one using historical controls) and none used a randomized design to be able to assess the comparative effectiveness of different interventions. Timing, duration and dose of treatment varied, and in the majority of studies patients were provided with other interventions which may have contributed to the reported outcomes. GRADE Tables are provided in Appendix S2.

### 3.5 Antiretroviral drugs as post‐exposure prophylaxis

Three studies reported a possible protective effect of LPV/r against coronavirus infection [38, 39, 40]. The first, a retrospective observational study from China, noted that 0 out of 19 patients hospitalized on same floor as SARS patients contracted the disease. Of the 19 patients, 11 were on differing regimens of antiretroviral therapy; none received LPV/r [38]. The second study, from South Korea, retrospectively enrolled health care workers considered at high risk of MERS infection. Of 22 healthcare workers given post‐exposure prophylaxis (PEP) comprising ribavirin and LPV/r, none were infected; this compared to 9 of 21 healthcare workers not given PEP who became infected [39]. The third study, from China, compared characteristics of 8 HIV‐positive individuals on different antiretroviral regimens who had contracted COVID‐19 infection with 1166 patients who had not been infected [40]. No statistically significant relationship was found between type of antiretroviral regimen and infection status. The certainty of the evidence across outcomes was again very low due to uncertainty due to limited sample size, lack of uniformity of regimens being used to treat patients, and lack of information regarding intensity of exposure (Appendix S2).

### 3.6 Registered clinical trials

Of 85 titles screened, 25 registered trials were identified that plan to assess the safety and efficacy of antiretrovirals – 20 assessing LPV/r (including 1 for the treatment of MERS and one for SARS, the rest for COVID‐19), two ritonavir, two darunavir and cobicistat and one tenofovir alafenamide fumarate. Estimated completion dates are from March 2020 to January 2022 (Appendix S3).

## 4 CONCLUSIONS

This systematic review identified two randomized trials and 21 observational studies provided clinical outcome data on the use of LPV/r for the treatment of COVID‐19, SARS and MERS. The randomized trials showed no clinical benefit, the observational studies were inconclusive, and the certainty of the body of evidence across all important outcomes was low or very low. Based on available evidence it is uncertain whether LPV/r and other antiretrovirals improve clinical outcomes in severe symptomatic disease or prevent infection among patients at high risk of acquiring COVID‐19. Any differences in potential therapeutic effect of LPV/r between SARS, MERS and COVID‐19 may partly be due to different clinical presentations; many of the patients had complicated courses including stays in intensive care units and were on multiple concurrent, unproven treatments.

Several randomized trials are planned to assess the safety and efficacy of antiretroviral drugs, including LPV/r, for the treatment of COVID‐19, MERS‐CoV and SARS‐CoV. While the conduct of such trials is challenging [41], high quality evidence is needed to improve clinical and programmatic decisions to use antiretroviral drugs for current and future coronavirus outbreaks.

The procurement and use of LPV/r or other antiretroviral drugs to treat or prevent COVID‐19 infection should take into consideration the need to ensure continued availability for people living with HIV who need LPV/r as part of their antiretroviral therapy. Overuse of LPV/r for COVID‐19 in the current epidemic runs a risk of shortage of a drug that is currently used as a second line treatment for people living with HIV.

WHO plans to update this review at least monthly throughout 2020, and longer as needed, to update the evidence as new studies are completed.

## Competing interests

The authors have no conflict of interest to declare.

## Authors’ contributions

NF and SN conceived the review. NF undertook all reviews and extracted the data, which was verified by AR. NF, AC, SN, AR, MV and MD interpreted the data. All authors contributed to the writing of the manuscript and approved the final version.

## Funding

This work was partly supported by a grant to the Bill & Melinda Gates Foundation.

### Disclaimer

The authors alone are responsible for the views expressed in this article and they do not necessarily represent the views of the organization.

## Supporting information


**Appendix S1. **Search terms.

**Appendix S2. **Grade assessment.

**Appendix S3. **Planned clinical trials of antiretroviral drugs.
