# Supplementary material for: Systematic review of the efficacy and safety of antiretroviral drugs against SARS, MERS or COVID‐19: initial assessment
Source: J Int AIDS Soc. 2020 Apr 1;23(4):e25489. doi: 10.1002/jia2.25489 (PMC7158851; doi:10.1002/jia2.25489)
Supplement: Supplementary file 1 — Appendix S1. Search terms. [file JIA2-23-e25489-s001.docx]

**Supplementary Appendix**

**1. Search terms**

**1.1 Search Strategy for Pubmed**

| #1 | “Sars Virus” OR SARS [TIAB] OR “Middle East Respiratory Syndrome Coronavirus”[MH] OR “mers virus” [TIAB] OR “mers-cov” [TIAB] OR “Middle East Respiratory Syndrome” [TIAB] OR “Severe Acute Respiratory Syndrome” [MH] OR “Severe Acute Respiratory Syndrome” [TIAB] OR ((Novel [TIAB] AND (coronavirus*[ TIAB] OR coronovirus*[ TIAB] OR “coronoravirus*”[ TIAB] OR “coronaravirus*”[TIAB] OR "Coronavirus"[Mesh] OR "Coronavirus Infections"[Mesh] )) OR "Wuhan coronavirus" [Supplementary Concept]) OR "2019?nCoV"[ TIAB]) OR “WN-CoV”[TIAB] OR nCoV[TIAB] OR "novel coronavirus"[ TIAB] OR “COVID-19”[MH] OR “COVID-19”[ALL] |
| --- | --- |
| #2 | Lopinavir [MH] OR 2494G1JF75 [RN] OR lopinavir[TW] OR aluvia[TW] OR kaletra[TW] OR lopinavir*[TW] |
| #3 | Tenofovir[MH] OR tenofovir[TW] OR viread[TW] OR TDF[TW] OR Truvada[TW] |
| #4 | Emtricitabine[MH] OR Emtricitabine[TW] OR Emtriva[TW] OR FTC[TW] |
| #5 | Nelfinavir[MH] OR nelfinavir[TW] OR viracept[TW] OR NFV[TW] |
| #6 | Indinavir[MH] OR Indinavir[TW] OR Crixivan[TW] |
| #7 | Saquinavir[MH] OR Saquinavir[TW] OR Fortovase[TW] OR Invirase[TW] |
| #8 | Lamivudine[MH] OR Lamivudine[TW] OR 3TC[TW] OR epivir[TW] |
| #9 | Zidovudine[MH] OR Zidovudine[TW] OR azidothymidine[TW] OR AZT[TW] OR ZDV[TW] OR Retrovir[TW] |
| #10 | #2 OR 3 OR #4 OR #5 OR #6 OR #7 OR #8 OR #9 |
| #11 | #1 AND #10 |

**1.2. Search Strategy for EMBASE**

| #1 | exp 'severe acute respiratory syndrome' OR 'severe acute respiratory syndrome':ti,ab,de OR covid OR 2019ncov OR sars OR exp 'middle east respiratory syndrome coronavirus' OR ncov OR ((exp 'pneumonia' OR pneumonia:ti,ab,de) AND wuhan:ti,ab,de) OR mers:ti,ab OR 'middle east respiratory syndrome':ti,ab,de OR (coronavirus:ti,ab,de AND wuhan:ti,ab,de) |
| --- | --- |
| #2 | Exp 'lopinavir' OR exp 'lopinavir plus ritonavir' OR exp 'lamivudine plus lopinavir plus ritonavir' OR '192725-17-0':rn OR aluvia:ti,ab,de OR kaletra:ti,ab,de OR lopinavir*:ti,ab,de |
| #3 | (exp 'tenofovir' OR 'tenofovir' OR pmpa:ti,ab,de OR '147127-19-3':rn) AND '147127-20-6':rn AND exp 'emtricitabine' OR 'emtricitabine' OR '137530-41-7':rn OR '143491-54-7':rn OR '143491-57-0':rn OR 'bw 524 w 91':ti,ab,de OR 'bw 524w':ti,ab,de OR 'bw 524w91':ti,ab,de OR 'bw524w':ti,ab,de OR 'bw524w91':ti,ab,de OR 'coviracil':ti,ab,de OR 'emtriva':ti,ab,de OR 'psi 5004':ti,ab,de OR 'psi5004':ti,ab,de OR 'racivir':ti,ab,de |
| #4 | ' exp nelfinavir' OR 'nelfinavir':ti,ab,de OR '159989-64-7':rn OR '159989-65-8':rn OR 'ag 1343':ti,ab,de OR 'ag 1346':ti,ab,de OR 'ag1343':ti,ab,de OR 'ag1346':ti,ab,de OR 'ly 312857':ti,ab,de OR 'ly312857':ti,ab,de OR 'nelfinavir mesilate':ti,ab,de OR 'nelfinavir mesylate':ti,ab,de OR 'viracept':ti,ab,de |
| #5 | 'exp indinavir' OR 'indinavir':ti,ab,de OR exp 'indinavir plus ritonavir' OR '150378-17-9':rn OR '157810-81-6':rn OR '180683-37-8':rn OR 'crixivan':ti,ab,de OR 'elvenavir':ti,ab,de OR 'indinavir monohydrate':ti,ab,de OR 'indinavir sulfate':ti,ab,de OR 'indinavir sulphate':ti,ab,de OR 'indivan':ti,ab,de OR 'indivir':ti,ab,de OR 'l 735524':ti,ab,de OR 'l735524':ti,ab,de OR 'mk 0639':ti,ab,de OR 'mk 639':ti,ab,de OR 'mk0639':ti,ab,de OR 'mk639':ti,ab,de |
| #6 | Exp 'saquinavir' OR exp 'ritonavir plus saquinavir' OR saquinavir:ti,ab,de OR '127779-20-8':rn OR '149845-06-7':rn OR 'ro 31 8959':ti,ab,de OR 'ro 31 8959 003':ti,ab,de OR 'ro 31-8959':ti,ab,de OR 'ro 31-8959-003':ti,ab,de OR 'ro31 8959':ti,ab,de OR 'ro31 8959 003':ti,ab,de OR 'ro31-8959':ti,ab,de OR 'ro31-8959-003':ti,ab,de |
| #7 | Exp 'lamivudine' OR lamivudine:ti,ab,de OR '134678-17-4':rn OR '134680-32-3':rn OR 'epivir':ti,ab,de OR 'epivir 3tc':ti,ab,de OR 'epivir hbv':ti,ab,de OR 'epivir-hbv':ti,ab,de OR 'gr 103665':ti,ab,de OR 'gr 109714x':ti,ab,de OR 'gr103665':ti,ab,de OR 'gr109714x':ti,ab,de OR 'hepivir':ti,ab,de OR 'heptodin':ti,ab,de OR 'heptovir':ti,ab,de OR 'inhavir':ti,ab,de OR 'ladiwin':ti,ab,de OR 'lamidac':ti,ab,de OR 'lamivir':ti,ab,de OR 'nsc 620753':ti,ab,de OR 'nsc620753':ti,ab,de OR 'slamivudine':ti,ab,de OR 'zeffix':ti,ab,de OR 'zefix':ti,ab,de OR exp'dolutegravir plus lamivudine plus tenofovir alafenamide' OR exp 'efavirenz plus lamivudine plus tenofovir disoproxil' OR exp 'efavirenz plus lamivudine plus zidovudine' OR exp 'lamivudine plus raltegravir' OR exp 'lamivudine plus stavudine' OR 'lamivudine plus zidovudine' |
| #8 | Exp 'zidovudine' OR '30516-87-1':rn OR 'adovi':ti,ab,de OR 'apo-zidovudine':ti,ab,de OR 'aviral':ti,ab,de OR 'avirzid':ti,ab,de OR 'azidodeoxythymidine':ti,ab,de OR 'azidomine':ti,ab,de OR 'azidothymidine':ti,ab,de OR 'azt':ti,ab,de OR 'aztec drug':ti,ab,de OR 'bio zt':ti,ab,de OR 'bw a 509 u':ti,ab,de OR 'bw a509u':ti,ab,de OR 'bwa509u':ti,ab,de OR 'novo-azt':ti,ab,de OR 'pranadox':ti,ab,de OR 'retrocar':ti,ab,de OR 'retrovir':ti,ab,de OR 'retrovir-azt':ti,ab,de OR 'thymidine 3 azido':ti,ab,de OR 'zdv':ti,ab,de OR 'zidis':ti,ab,de OR 'zidovir':ti,ab,de OR 'zydowin':ti,ab,de |
| #9 | #2 OR #3 OR #4 OR #5 OR #6 OR #7 OR #8 |
| #10 | #1 AND #9 |
