# Supplementary material for: Systematic review of the efficacy and safety of antiretroviral drugs against SARS, MERS or COVID‐19: initial assessment
Source: J Int AIDS Soc. 2020 Apr 1;23(4):e25489. doi: 10.1002/jia2.25489 (PMC7158851; doi:10.1002/jia2.25489)
Supplement: Supplementary file 2 — Appendix S2. Grade assessment. [file JIA2-23-e25489-s002.docx]

**2. Grade Assessment**

**2.1. Treatment**

| **Quality assessment** | | | | | | **Patients** | **Effect** | **Certainty** | |
| --- | --- | --- | --- | --- | --- | --- | --- | --- | --- |
| **No of studies** | **Design** | **Risk of bias** | **Inconsistency** | **Indirectness** | **Imprecision** |  |  |  |  |
| **MERS** | | | | | | | | | |
| 6 observational studies | 2 retrospective cohorts and 4 case reports | High^1^ | Low | Low | High^2^ | 165 | 42/165 died | Very low |  |
| **SARS** | | | | | | | | | |
| 3 observational studies | Observational studies | High^1^ | Low | Low | High^2^ | 117 | 5/117 died | Very low |  |
| **COVID-19 – randomized trials** | | | | | | | | | |
| 2 randomized trials | Randomized trials | Moderate^3^ | Not relevant | Low | High^2^ | 199 | RR 0.62  (0.27-1.43)* | Low |  |
| **COVID-19 – observational studies** | | | | | | | | | |
| 10* | 6 retrospective cohorts, 1 case series and 3 case reports | High^1^ | Low | Low | High^2^ | 361 | 3/361 died | Very low |  |

1. Retrospective observational studies with varying baseline characteristics, including disease severity; timing, duration and dose of treatment varied; several studies provided co-interventions which may have contributed to the reported outcomes

2. Small sample size

3. investigators not blinded to the intervention (no placebo)

* Data from trial of severe cases only; data not pooled with trial including only mild cases

** data not included for 1 study (PMID 32171866) because survival could not be linked to receipt of intervention

**2.2. Post-exposure prophylaxis**

| **Quality assessment** | | | | | | **Patients** | **Effect** | **Certainty** |
| --- | --- | --- | --- | --- | --- | --- | --- | --- |
| **No of studies** | **Design** | **Risk of bias** | **Inconsistency** | **Indirectness** | **Imprecision** |  |  |  |
| **MERS** | | | | | | | | |
| 1 | 1 matched cohort study and 3 case reports | High^1^ | Low | Low | High^2^ | 19 | 0 infections | Very low |
| **SARS** | | | | | | | | |
| 1 | Observational studies | High^1^ | Low | Low | High^2^ | 123 | 0 infections | Very low |
| **COVID-19** | | | | | | | | |
| 1 | Observational studies | High^1^ | Low | Low | High^2^ | 8 | 0 infections | Very low |

1. Retrospective observational studies; variability in drugs provided; lack of information regarding intensity of exposure.

2. Small sample size
