# Supplementary material for: Systematic review of the efficacy and safety of antiretroviral drugs against SARS, MERS or COVID‐19: initial assessment
Source: J Int AIDS Soc. 2020 Apr 1;23(4):e25489. doi: 10.1002/jia2.25489 (PMC7158851; doi:10.1002/jia2.25489)
Supplement: Supplementary file 3 — Appendix S3. Planned clinical trials of antiretroviral drugs. [file JIA2-23-e25489-s003.docx]

**3. Planned clinical trials of antiretroviral drugs**

|  | **Clinical Trials Identifier** | **Title** | **Participants** | **Sponsor** | **Status** | **Estimated completion** | **Intervention** |
| --- | --- | --- | --- | --- | --- | --- | --- |
| 1 | NCT04295551 | Multicenter clinical study on the efficacy and Safety of Xiyanping injection in the treatment of new Coronavirus infection pneumonia (general and severe) | 80 | Jiangxi Qingfeng Pharmaceutical Co. Ltd | Not yet recruiting | 14/07/2020 | Lopinavir/ritonavir tablets combined with Xiyanping injection vs Lopinavir/ritonavir treatment |
| 2 | NCT04291729 | Evaluation of Ganovo (Danoprevir) combined with Ritonavir in the treatment of novel coronavirus Infection (non randomized) | 50 | Ascletis Pharmaceuticals Co., Ltd., The Ninth Hospital of Nanchang | Recruiting | 31/03/2020 | Drug: Ganovo+ritonavir+/-Interferon atomization  Drug: Long acting interferonDrug: Recombinant cytokine gene-derived protein  Drug: Lopinavir+ritonavir  Drug: Chinese medicines +interferon atomization |
| 3 | NCT04286503 | The Clinical study of Carrimycin on treatment patients with COVID-19 | 520 | Beijing YouAn Hospital | Not yet recruiting | 28/02/2021 | Drug: Carrimycin  Drug: lopinavir/ritonavir tablets or Arbidol or chloroquine phosphate  Drug: basic treatment |
| 4 | NCT04276688 | Lopinavir/ ritonavir, ribavirin and IFN-beta combination for nCoV Treatment | 70 | The University of Hong Kong | Recruiting | 31/01/2022 | Drug: Lopinavir/ritonavir  Drug: Ribavirin  Drug: Interferon Beta-1B |
| 5 | NCT04275388 | Xiyanping Injection for the treatment of New Coronavirus infected pneumonia | 348 | Jiangxi Qingfeng Pharmaceutical Co. Ltd. | Not yet recruiting | 14/05/2020 | Drug: Xiyanping injection  Drug: Lopinavir / ritonavir, alpha-interferon nebulization |
| 6 | NCT04261270 | A randomized, open, controlled clinical study to evaluate the efficacy of ASC09F and ritonavir for 2019-nCoV Pneumonia | 60 | Tongji Hospital | Not yet recruiting | 01/07/2020 | Drug: Ritonavir+Oseltamivir |
| 7 | NCT04252885 | A randomized, open-label, controlled study of the efficacy of lopinavir plus ritonavir and arbidol for treating patients with novel coronavirus infection | 125 | Guangzhou 8th People's Hospital | Recruiting | 31/07/2020 | 50 cases: ordinary treatment plus of lopinavir (200mg) and ritonavir (50mg)  50 cases: ordinary treatment plus arbidol (100mg) (oral, tid, 200mg each time, taking for 7-14 days).  25 cases: given ordinary treatment. |
| 8 | NCT00578825 | A multi-centre, double-blinded, randomized, placebo-controlled trial on the efficacy and safety of lopinavir/ritonavir plus ribavirin in the treatment of severe acute respiratory syndrome | 340 | Hospital Authority, Hong Kong | Status unknown | Last updated in 2013 | Lopinavir/ritonavir plus ribavirin  In adults |
| 9 | NCT04255017 | A Prospective, randomized controlled clinical study of antiviral therapy in the 2019-nCoV Pneumonia | 400 | Tongji Hospital | Not yet recruiting | 01/06/2020 | Drug: Abidol hydrochloride  Drug: Oseltamivir  Drug: Lopinavir/ritonavir |
| 10 | NCT04261907 | Randomized open-label trial evaluating and comparing the safety and efficiency of ASC09/ritonavir and lopinavir/ritonavir for novel coronavirus lnfection | 160 | First Affiliated Hospital of Zhejiang University | Not yet recruiting | 30/06/2020 | ASC09/ritonavir vs lopinavir/ritonavir |
| 11 | NCT02845843 | MERS-CoV lnfection treated with a combination of lopinavir /ritonavir and interferon Beta-1b (MIRACLE). placebo-controlled double-blind randomized controlled trial | 194 | King Abdullah International Medical Research Center | Recruiting | 12/2020 | Lopinavir/ritonavir and Interferon beta-1b vs placebo |
| 12 | NCT04251871 | Treatment and prevention of traditional Chinese medicines (TCMs) on 2019-nCoV Infection: a prospective, open-labelled, randomized, controlled trial | 150 | Beijing 302 Hospital | Recruiting | 22/01/2021 | Drug: conventional medicines (oxygen therapy, alfa interferon via aerosol inhalation, and lopinavir/ritonavir) and Traditional Chinese Medicines (TCMs) granules  Conventional medicines: oxygen therapy, antiviral therapy (alfa interferon via aerosol inhalation, and lopinavir/ritonavir, 400mg/100mg, p.o, bid) for 14 days.  Traditional Chinese Medicines (TCMs) granules: 20g, p.o, bid, for 14 days.  Vs  Drug: Conventional medicines (oxygen therapy, alfa interferon via aerosol inhalation, and lopinavir/ritonavir)  Conventional medicines: oxygen therapy, antiviral therapy (alfa interferon via aerosol inhalation, and lopinavir/ritonavir, 400mg/100mg, p.o, bid) for 14 days. |
| 13 | NCT04252274 | Randomized trial of the efficacy and safety of darunavir and cobicistat for treatment of pneumonia caused by 2019-nCoV (DACO-nCoV) | 30 | Shanghai Public Health Clinical Center | Not yet recruiting | 31/12/2020 | Darunavir and cobicistat one tablet per day for 5 days, in addition to conventional treatments |
| 14 | ChiCTR2000030535 | Multi-Center Clinical Study on the Treatment of Patients with Novel Coronavirus Pneumonia (COVID-19) by Ebastine | 100 | Wuhan Red Cross Hospital,  Mianyang Central Hospital | Recruiting | NS | ebastine 10mg bid, interferon-alpha aerosol inhalation 5million U bid and Lopinavir 200 mg, 2 capsules at a time, bid |
| 15 | ChiCTR2000029308 | A randomized, controlled open-label trial to evaluate the efficacy and safety of lopinavir-ritonavir in hospitalized patients with novel coronavirus pneumonia (COVID-19) | 160 | Wuhan Jinyintan Hospital (Wuhan Infectious Diseases Hospital) | Recruiting | NS | Lopinavir-ritonavir tablets (each containing 200 mg of lopinavir and 50 mg of ritonavir), twice a day, 2 tablets at a time |
| 16 | ChiCTR2000030472 | An open and controlled clinical study to evaluate the efficacy and safety of Ganovo combined with ritonavir in the treatment of novel coronavirus pneumonia (COVID-19) | 20 | Shenyang Sixth People's Hospital, Chinese Govt. | Recruiting | NS | Ganovo/ ritonavir oral+conventional treatment |
| 17 | ChiCTR2000030187 | Clinical study for Lopinavir and Ritonavir in the treatment of novel coronavirus pneumonia (COVID-19) | 60 | Jingzhou First People's Hospital | Not yet recruiting | NS | Lopinavir and Ritonavir Tablets |
| 18 | ChiCTR2000030166 | Randomized, parallel control, open trial for Qing-Wen Bai-Du-Yin combined with antiviral therapy in the treatment of novel coronavirus pneumonia (COVID-19) | 20 | The 5th Medical Center Chinese PLA General Hospital | Not yet recruiting | NS | (Integrated Chinese and western medicine): Lopinavir-ritonavir tablet combined with recombinant human interferon alpha 2b injection(routine treatment)+Qing-Wen Bai-Du-Yin formula granules |
| 19 | ChiCTR2000030113 | Randomized controlled trial for safety and efficacy of Favipiravir in the treatment of novel coronavirus pneumonia (COVID-19) with poorly responsive ritonavir/ritonavir | 30 | The Third People's Hospital of Shenzhen | Recruiting | NS | Ritonavir/ritonavir (control) |
| 20 | ChiCTR2000029741 | Efficacy of Chloroquine and Lopinavir/ Ritonavir in mild/general novel coronavirus (CoVID-19) infections: a prospective, open-label, multicenter randomized controlled clinical study | 112 | Sun Yat-Sen University | Recruiting | NS | Ritonavir/ritonavir (control) |
| 21 | ChiCTR2000029573 | A multicenter, randomized, open-label, positive-controlled trial for the efficacy and safety of recombinant cytokine gene-derived protein injection combined with abidole, lopinavir/litonavir in the treatment of novel coronavirus pneumonia (COVID-19) patients | 600 | The First Affiliated Hospital of Medical College of Zhejiang University | Not yet recruiting | NS | Lopinavir/ritonavir 500mg/100mg/ time, p.o.tid.  Novaferon injection 20g/ time, atomized inhalation, tid.Lopinavir/litonavir 500mg/100mg/ time, p.o.tid. |
| 22 | ChiCTR2000029548 | Randomized, open-label, controlled trial for evaluating of the efficacy and safety of Baloxavir Marboxil, Favipiravir, and Lopinavir-Ritonavir in the treatment of novel coronavirus pneumonia (COVID-19) patients | 30 | The First Affiliated Hospital, Zhejiang University School of Medicine | Pending | NS | C:Lopinavir-Ritonavir: 2# (200mg/50 mg), twice daily, for 14days.; |
| 23 | ChiCTR2000029541 | A randomised, open, controlled trial for darunavir/cobicistat or Lopinavir/ritonavir combined with thymosin a1 in the treatment of novel coronavirus pneumonia (COVID-19) | 100 | Zhongnan Hospital of Wuhan University | Pending | NS | DRV/c group:DRV/c (800mg/150mg QD) + Conventional treatment containing thymosin (1.6 mg SC QOD) ;  LPV/r group:LPV/r (400mg/100mg bid) + Conventional treatment containing thymosin (1.6 mg SC QOD);other group |
| 24 | ChiCTR2000029539 | A randomized, open-label study to evaluate the efficacy and safety of Lopinavir-Ritonavir in patients with mild novel coronavirus pneumonia (COVID-19) | 328 | Tongji Hospital, Tongji Medical College, Huazhong University of Science and Technology | Recruiting | NS | experimental group:conventional standardized treatment and Lopinavir-Ritonavir;control group:  Conventional standardized treatment; |
| 25 | ChiCTR2000029468 | A real-world study for lopinavir/ritonavir (LPV/r) and emtritabine (FTC) / Tenofovir alafenamide Fumarate tablets (TAF) regimen in the treatment of novel coronavirus pneumonia (COVID-19) | 120 | Department of science and technology of sichuan province (2020YFS0006), Institute of Emergency Medicine and Disaster Medicine Sichuan People's Hospital, Sichuan Academy of Medical Sciences | Pending | NS | experimental group:Lopinavir/litonavir (LPV/r)+ emtritabine (FTC)/ Tenofovir alafenamide Fumarate tablets (TAF) in combination;  Historical Control:LPV/r; |

**Summary**

| **Drug** | **Studies** | **Patients** |
| --- | --- | --- |
| LPV/r | 19 | 3897 |
| LPV | 1 | 100 |
| /r | 2 | 80 |
| DRV/COBI | 2 | 130 |
| TAF/3TC | 1 | 120 |
